# T-Cell Clustering in Neoplastic Follicles of Follicular Lymphoma

**DOI:** 10.1007/s12307-018-0217-1

**Published:** 2018-09-11

**Authors:** Patrick Schnotalle, Karoline Koch, Rex K. H. Au-Yeung, Sarah Reinke, Karsten Winter, Markus Loeffler, Ulf-Dietrich Braumann, Wolfram Klapper

**Affiliations:** 1 Institute for Medical Informatics, Statistics and Epidemiology (IMISE), Medical Faculty, University of Leipzig, Leipzig, Germany; 2 Department of Pathology, Hematopathology Section and Lymph Node Registry, University of Kiel/University Hospital Schleswig-Holstein, Arnold-Heller-Strasse 3, Haus 14, 24105 Kiel, Germany; 3 Institute of Anatomy, Medical Faculty, University Leipzig, Leipzig, Germany; 4 Faculty of Electrical Engineering and Information Technology, Leipzig University of Applied Science (HTWK), Leipzig, Germany; 5 Fraunhofer Institute for Cell Therapy and Immunology (IZI), Leipzig, Germany

**Keywords:** Follicular lymphoma, Microenvironment, Image analysis, Point pattern analysis; PD1

## Abstract

The nonneoplastic microenvironment is abundant in follicular lymphoma. Its composition has been reported to be associated with the course of the disease. Lack of animal models hampers studies of interaction between lymphoma and bystander cells. We aimed to identify indicators of cellular interaction exemplified by nonrandom distribution of cell types within neoplastic follicles. Physiological germinal centers and follicles in follicular lymphoma were stained to identify macrophages, all T, follicular T-helper, dendritic and B cells. Density of cell types and cell distribution (spatial point pattern) were analyzed by digital image analysis. The density of all T, follicular T-helper and dendritic cells was higher in the dark zone than in the light zone of physiological germinal centers. Densities of cell types in follicular lymphoma were intermediate between the light and the dark zone. All cell types analyzed showed a completely random spatial distribution pattern within the dark and the light zone, respectively. In follicular lymphoma B cells and macrophages displayed complete spatial randomness. In contrast, all T cells, follicular T-helper cells and dendritic cells showed clustering of each individual cell type within a radius of 6–10 μm in the lymphoma. We conclude that the distribution of nonneoplastic cells within follicles of follicular lymphoma is not random. T cells and dendritic cells form clusters within the follicles, suggestive of sites of interaction between microenvironment and lymphoma cells. These clusters might help to understand the interaction of lymphoma cells with the microenvironment and might provide a structure for therapeutic intervention.

**Electronic supplementary material:**

The online version of this article (10.1007/s12307-018-0217-1) contains supplementary material, which is available to authorized users.

## Introduction

Follicular lymphoma (FL) is the second most common B cell lymphoma and represents about 20% of all lymphomas in Central Europe [1]. Whereas the pathogenesis of FL is initiated during B cell maturation in the bone marrow, in many aspects the fully developed lymphoma reflects a developmental stage of germinal center (GC) B cells [2]. FL mimic the GC in their immunophenotype (expression of GC markers like CD10 and BCL6), cytomorphology (centroblasts and centrocytes) and growth pattern (follicular architecture). Furthermore, FL harbor GC-specific nonneoplastic bystander cells (Klapper, 2011). This microenvironment is composed of relatively widely distributed cell types such as macrophages and T cells. In addition, the GC microenvironment contains immune cells that are virtually confined to GC, such as follicular T-helper cells (T_FH_) and follicular dendritic cells (FDC) [3]. This microenvironment in FL is frequently so abundant that nonneoplastic bystander might outweigh neoplastic cells within affected lymph nodes.

The composition of the microenvironment in FL as assessed by gene expression profiling has been shown to be associated with the clinical course of the disease (Dave et al., 2004). Studies trying to translate the gene expression data into surrogate immunohistochemical markers revealed discrepant results regarding the prognostic impact of content and localization of bystander cells (de Jong et al., 2009). Published studies have assessed the content of bystander cell subtypes within lymphoma biopsy specimens using visual inspection of image analysis. However, there is little data on the role of the microenvironment beyond the descriptive level of cell content. The reason for this is the lack of animal models, the inability to maintain primary FL cells in culture without phenotypic alterations and the difficulties in applying live-cell imaging to human lymphoma biopsy specimens. However, growing evidence suggests that bystander cells influence lymphoma B cells, and vice versa, via paracrine signaling or via B cell receptor dependent signaling [4–7]. Interaction between FL and bystander cells can also be deduced from observations on patient specimens, since the transition from a follicular to a diffuse growth pattern is accompanied by changes in the microenvironment, e.g. a loss of the follicular dendritic cell (FDC) component [8].

We hypothesized that a quantitative analysis of spatial arrangements of B cells and microenvironmental bystander cells would provide insights into potential interactions of cell types, e.g. by identifying a nonrandom distribution of cells (clustering). We used a newly developed image processing chain for fluorescence multi-stainings to analyze spatial cell distributions in benign and malignant lymph follicles.

## Materials and Methods

### Fluorescence Multi-Stainings and Image Acquisition

FFPE tissue from tonsils and FL were obtained from the files of the Department of Pathology, University Hospital Schleswig-Holstein, Campus Kiel. The use of tissue was in accordance with the Helsinki Declaration and the guidelines of the internal ethics review board of the medical faculty of the University of Kiel (statement number 447/10).

Antigen retrieval was performed on FFPE sections by pressure cooking for 3 min at pH 6 in a citrate-based buffer. Primary antibodies were applied simultaneously for an hour at room temperature. On each section, three primary antibodies were applied: Pax5 to stain B cell nuclei, Ki67 to highlight the proliferative compartment and one membrane bound marker highlighting a bystander cell type (CD3 for T cells, PD1 for follicular T-helper cells, CD21 for follicular dendritic cells or CD68 for macrophages). The species-specific, fluorescence-labeled secondary antibodies (Alexa Fluor 488 donkey anti-goat, Alexa Fluor 555 donkey anti-rabbit/anti-mouse and Alexa Fluor 647 donkey anti-rabbit/anti-mouse) were applied simultaneously for 1 h at room temperature. All antibodies are listed in supplementary Table 1. Nuclear counterstaining was performed using diamidino-phenylindole (DAPI).

Epifluorescence microscopy images of three follicles per section were captured using the software *VisiView* (version 1.7.2, Visitron Systems GmbH, Puchheim, Germany), controlling a 12bit monochrome camera (1.6 Megapixels), Spot RT Slider (Diagnostic Instruments), mounted on an Axioplan2 (Zeiss) with an EC Neofluar ×10 (Zeiss). The digital images exhibit a pixel sampling grid of 0.74 μm with respect to the original specimen size. Delineation of the dark and light zones in the physiological follicles was done by visual inspection on the basis of the Ki67 and DAPI staining.

### Image Preprocessing

Unspecific channel-wise preprocessing was accomplished using *Mathematica* (version 11.0.1.0, Wolfram Research Inc., Urbana Champaign, IL, USA) in order to further improve the quality of the fluorescence images. Additionally, total variation denoising [9] was applied using a Poisson statistics model [10] to reduce fluorescence imaging noise.

### Image Processing for Cell Segmentation

The segmentation of cell nuclei or cell membranes was accomplished using individual cell type-based processing chains, which in turn were implemented in *Mathematica*. A detailed description of the method is given in the supplementary data.

### Cell Count and Density

For each point pattern the total number of points (n) and the point density (n/total image area) were calculated using R (version 3.2.2, The R Foundation for Statistical Computing, Vienna, Austria) and RStudio (Version 1.0.44, https://www.rstudio.com/). Densities were visualized using density plots in which the spatial distribution and the local accumulation or absence of points in point patterns can be perceived (supplementary Fig. 1). Differences in densities were calculated by t-test.

### Functional Statistics of Cell Distribution Pattern

To assess the distribution of microenvironmental cell types within the physiological and neoplastic follicles we used Ripley’s *K*- function [11], which distinguishes complete spatial randomness (CSR), clustering and regularity. Analysis was performed using R. For details see Supplementary methods.

## Results

### Density of B Cell and Microenvironmental Cells

Digital image analysis was applied to physiological GC in tonsil tissue (*n* = 3) and FL (n = 3). Three follicles were analyzed in each tissue specimen (total of *n* = 9 physiological and n = 9 malignant follicles). Supplementary Figs. 2 and 3 illustrate the image segmentation procedure developed for the current project for a healthy follicle and a malignant follicle in an FL, respectively. In physiological GC multi-staining for Ki67 allowed the identification of a highly proliferative dark and a low proliferating light zone by visual inspection, and the analysis of densities was performed separately in the two zones (supplementary Fig. 2). FL lacks compartmentalization into a dark and light zone. Thus, FL follicles were analyzed in total.

**Fig. 1:**
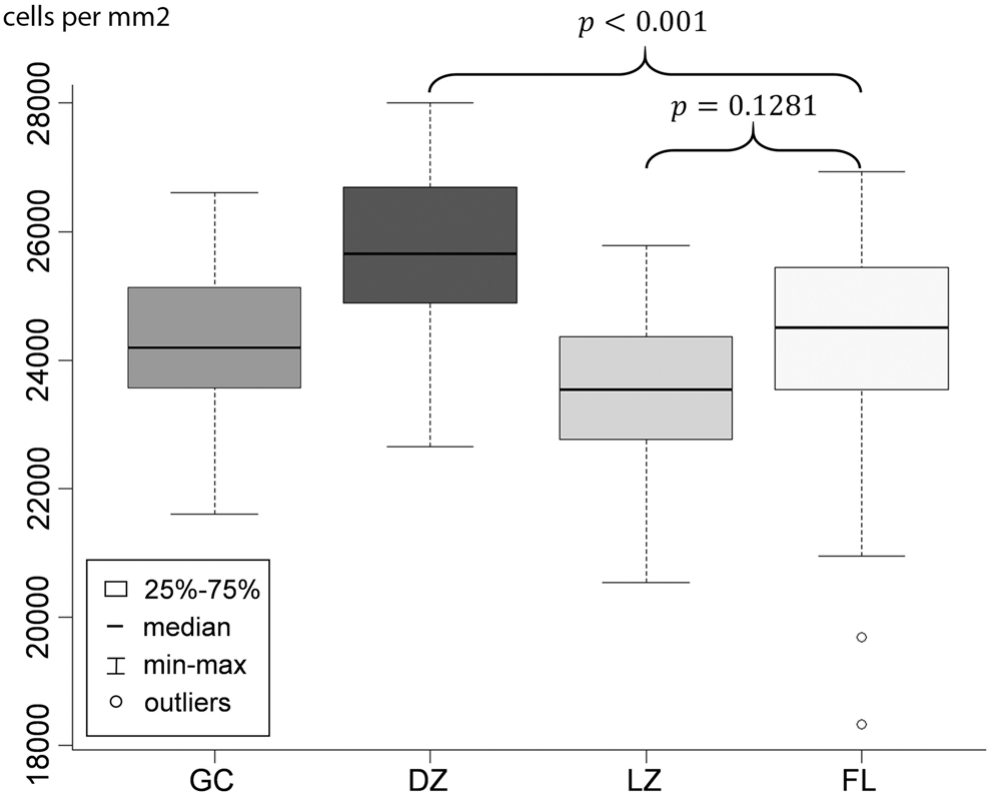
Density of the B cells in the whole germinal center (GC), the light zone (LZ), the dark zone (DZ) and in follicular lymphoma (FL). Density is given on the y-axis as cells per mm^2^. *P*-values according to t-test

To understand the accessibility of B cells to accessory cells, such as FDC and T cells, in physiological and neoplastic cells in malignant follicles, we determined the density (cells per mm^2^) of cell types within physiological GC and FL follicles. Consistent with the morphological impression, the B cell density was higher in the dark zone than in the light zone of physiological GC (*p* < 0.001, Fig. 1). In FL the B cell density was significantly lower than in the dark zone (*p* < 0.001, Fig. 1) and slightly higher than in the light zone of physiological GC (*p* = 0.1281, Fig. 1).

**Fig. 2:**
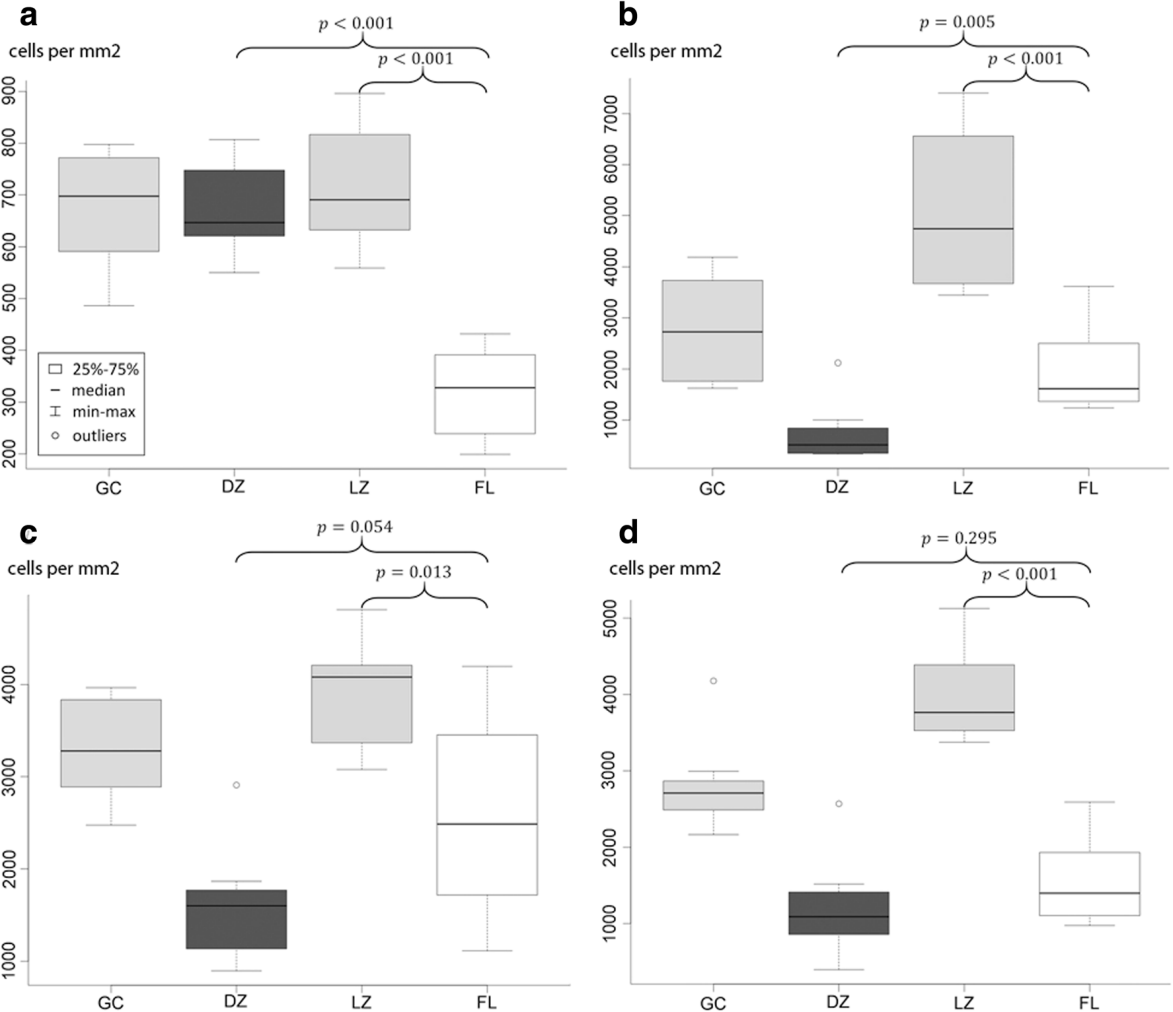
The densities of the bystander cells in whole physiological germinal centers (GC), the light zone (LZ), the dark zone (DZ) and in follicular lymphoma (FL). **a** macrophages identified by CD68, **b** follicular dendritic cells identified by CD21, **c** all T cells identified by CD3, **d** T_FH_ identified by PD1. Density on the y-axis is given as cells per mm^2^. P-values according to t-test

As expected, the densities of T cells (CD3), T_FH_ (PD1) and FDC (CD21) were higher in the light zone than in the dark zone of physiological GC (*p* < 0.001, p < 0.001 and p < 0.001, respectively, Fig. 2). Although on visual inspection starry sky macrophages predominantly occur in the dark zone of the GC, the cell density of macrophages did not differ significantly between the dark and the light zone (*p* = 0.42 Fig. 2). The densities of all T cells, T_FH_ and FDC in FL were lower than in the light zone (*p* = 0.013, *p* < 0.001 and p < 0.001, respectively, Fig. 2) but higher than in the dark zone (*p* = 0.054, *p* = 0.295 and *p* = 0.005, respectively, Fig. 2). In contrast, the density of macrophages was significantly lower in FL than in both the dark zone and the light zone of the physiological GC (p < 0.001 and p < 0.001, respectively, Fig. 2).

### Distribution Pattern of B Cells and Microenvironmental Cells within the Follicles

**Fig. 3:**
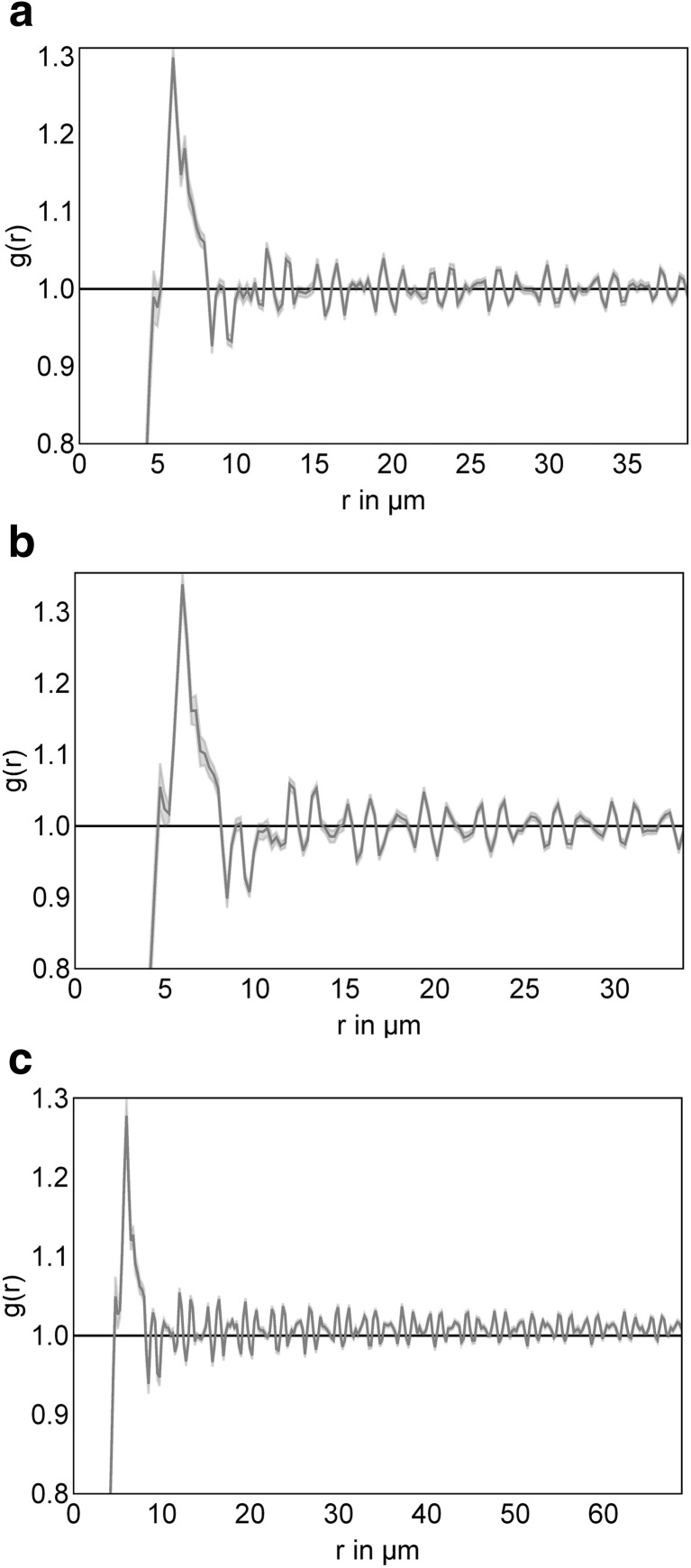
The distribution pattern of B cells within the light zone (LZ) (**a**), dark zone (DZ) (**b**) and FL (**c**). The results of the functions g(r) is indicated on the y-axis. The thick line represents the median of pooled analysis of g(r) in the three specimens (*n* = 9 for LZ (**a**), DZ (**b**) or FL (**c**)). Values below 1 suggest regularity, values around 1 complete spatial randomness and vlaues above 1 clustering. The values are given for each radius around the selected target cell (X-axis: distance r in μm). Thus, the course of the line and the envelope differs dependent of the radius around the target cell. The envelopes in light grey indicate the minimum and the maximum of all follicles but are very narrow in this analysis due to the homogeneous results

**Fig. 4:**
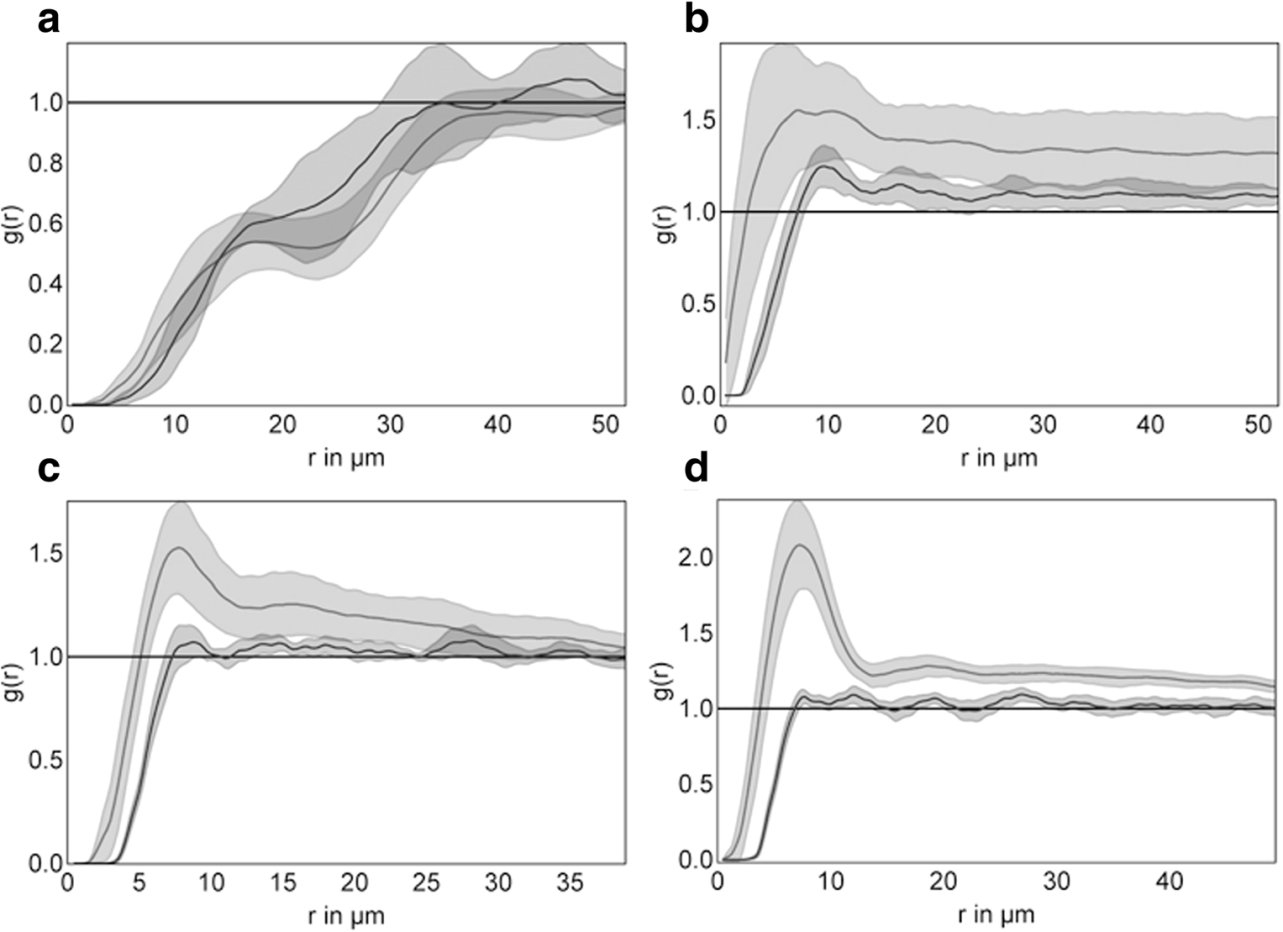
Point-pattern analysis of the distribution of bystander cells within follicles of follicular lymphoma (FL) and the light zone of the physiological germinal centers (LZ). Pooled analysis of all follicles in each of the three specimens (n = 9). X-axis: distance r in μm, Y-axis: g(r) functions. The computed envelope for FL (light grey areas) and the computed envelope for LZ (dark grey areas indicate the minimum and the maximum and the thick line represents the median of all functions. **a** macrophages identified by *CD68*, (**b**) follicular dendritic cells identified by CD21, (**c**) all T cells identified by CD3, (**d**) T_FH_ identified by PD1

In order to understand whether the neoplastic follicles in FL have a spatial organization that is not detectable by visual inspection, we applied point pattern analysis to identify the distribution of a given cell type as random (complete spatial randomness), regular or clustered within the neoplastic follicles (supplementary Fig. 4 shows virtual examples of these distribution patterns and the results of point pattern analysis indicated by the function g(r)). This method identifies clustering of all T cells, T_FH_ and FDC within the light zone when applied to the whole physiological GC (data not shown). Since our aim was to identify the spatial organization within areas that appeared homogeneous on visual inspection, the method was applied to the light and dark zone and to malignant FL follicles separately. At first we tested the distribution pattern of B cells, which appeared to be completely spatially random in FL as well as in the light and dark zones of the GC (g(r) close to 1, Fig. 3). Similarly, CD68-positive macrophages showed complete spatial randomness within FL as well as in the light zone with a radius of >40 μm (g(r) = 1, Fig. 4) and dark zone of GC (data not shown).

Differences in the distribution pattern between the light zone on the one hand and FL on the other hand became evident for all T cells (CD3), T_FH_ (PD1) and FDC (CD21). Within the dark zone and the light zone all of the cell types analyzed showed a distribution close to complete spatial randomness (g(r) close to 1, Fig. 4). In contrast, T cells, T_FH_ (PD1) and FDC within FL clustered (g(r) >1, Fig. 4). This clustering is indicated by a deviation of the function g(r) to values above 1. As indicated in Fig. 4, a bending of the curve and a deviation from the value 1 is most evident in a radius of 5–10 μm around a given cell of the respective cell type. Since deviation to values above 1 indicates clustering, the analysis suggests that within a radius of 5–10 μm around a given cell type, the number of other cells of the same type is significantly increased. This phenomenon of clustering was observed for CD3 positive T cells, T_FH_ and FDC in FL (Fig. 4). We were able to confirmed the findings for PD1 clustering in an independent cohort of *n* = 9 FL (supplementary Fig. 5 and supplementary methods).

## Discussion

FL is an indolent B cell lymphoma with a tissue architecture mimicking the assumed physiological counterpart of the lymphoma, the GC. Whereas GC show a zonation in dark and light zones with differing cellular composition and function [12], FL follicles are considered to be disorganized [8]. However, recent data shows that FL B cells depend on paracrine and receptor-dependent interaction with T cells and macrophages for proliferation and survival [4–7]. These data suggest that the arrangement of B cells and the microenvironment in FL is not random. However, the tissue architecture in FL may be more complex and not ascertainable by visual inspection. Previously published point pattern approaches for the image analysis of FL mainly focused on the analysis of the cell types found in FL [13, 14]. We add a new aspect to the digital image analysis of FL by assessing the toponomics and local relationships of different cell populations within the neoplastic follicles. This study exemplifies the potential of advanced histometrical methods. Because of the high B cell density in the dark zone of the GC, macrophages are easily identifiable on visual inspection as tingible body macrophages, but they are less apparent on visual inspection in the light zone. However, our analysis indicates that the macrophage density in the light zone is similar to that in the dark zone.

In this study we were able to demonstrate that T cells and FDC cluster within small areas of 6-10 μm within the neoplastic follicle. These clusters may constitute an interaction between the bystander cell subtypes themselves. However, it seems more likely that the clusters of T cells represent areas of interaction with neoplastic cells. A similar phenomenon was described recently by Pangault and co-workers, who found phosphorylated STAT6 expression in lymphoma cells in close proximity to T cells expressing PD1 [6]. Staining for PD1 is a commonly used technique in histology analysis to identify T_FH_. However, PD1 expression is not entirely specific for T_FH_ and we cannot rule out that other cells but T_FH_ are clustering [15, 16]. To further characterize this cell population, different combinations of multistainings are necessary. However, T_FH_ are a cell population of specific interest in FL since their abundance in the microenvironment has been associated with clinical outcome [16–18] and PD1 and its ligands present potential therapeutic targets.

Our study will require confirmation and needs to be extended in future studies. Since we only analyzed a small number of malignant follicles, future studies will need to identify the variability of this phenomenon in different patients and investigate whether it is correlated with clinical, pathological or genetic features of the lymphoma. Furthermore, the extent and composition of the microenvironment in FL is variable and might be correlated with clustering. Thus clustering needs to be studied in a larger cohort of patients. Multiplexing of immunohistochemical staining in future studies will help to determine whether clusters of T cells and FDC overlap. And finally, combining the identification of biomarkers of B cell activation, such as expression of phosphorylated STAT6 or cell proliferation in proximity to clusters of bystander cells, will provide further insights into the biological relevance of the phenomenon observed in our study. If the clusters of bystander cells prove to be areas of active lymphoma-microenvironment interaction, these clusters might also be targets of immune-checkpoint therapy.

## Electronic Supplementary Material

ESM 1 (DOCX 3708 kb)
