# Supplementary material for: T-Cell Clustering in Neoplastic Follicles of Follicular Lymphoma
Source: Cancer Microenviron. 2018 Sep 11;11(2-3):135–40. doi: 10.1007/s12307-018-0217-1 (PMC6250612; doi:10.1007/s12307-018-0217-1)
Supplement: Supplementary file 1 — (DOCX 3708 kb) [file 12307_2018_217_MOESM1_ESM.docx]

**Supplementary data**

**Supplementary methods**

Image processing for cell segmentation

B cell nuclei were detected on the basis of *Pax5* IHC fluorescence staining using Bernsen filtering (Bernsen 1986). Subsequent object separation was accomplished using a sequence of distance transform within segmented objects followed by local maxima detection therein (with a certain minimum protrusion to really catch the places of cell centers in the Bernsen result) using a Laplacian filter (to obtain the 2^nd^ derivative). Such local maxima denote the centers of assumed distinct objects. Using another distance transform mutual distances between these assumed object centers were computed. A sequence of smoothing followed by a Laplacian filter ( (Marr 1980), also named Laplacian of Gaussian) finally found those lines, which in turn were used as separators within incompletely separated B cells.

T cells stained using *CD3* and follicular T-helper cells stained using *PD1* were detected using a morphological top-hat transform (Meyer 1996), which basically reveals a difference between the scalar fluorescence image and the opening of the same image, which in turn is being computed, using a structuring element as dilation of the erosion of the image. Unlike for B cell segmentation, due to the *CD3* membrane staining, T cells can occur either as annulus-like structures or as portions of closed membrane parts. As top-hat transform emphasizes objects which are both brighter than their neighborhood and are smaller than the structuring element, we can assume that both facets of *CD3*-labeled T cells are being detected. The top-hat transform was followed by a global thresholding using an intra-class variance-minimization based binarization method (Otsu 1979). As soon as the resulting segments were larger than a typical size for a single cell, object separation using the Marr-Hildreth operator-based approach (see above) was applied.

Follicular dendritic cells (FDCs), which were membrane-stained using *CD21,* were segmented by means of Otsu’s image-global method (see above). FDCs occur in a large variety of shapes in 2D. Methods from mathematical morphology (as used for T cells) cannot be used appropriately here. Also, local thresholding (as used for B lymphocyte nuclei) is not appropriate here, as *CD21* images exhibit quite high noise. Similar to the B/T lymphocyte segmentation, an object separation procedure based on the Marr-Hildreth-operator was applied (see above). As FDCs develop a network, respective processes are basically also *CD21* positive, however, the intensity is lower than at the soma membrane of FDCs; hence the segmentation is restricted to the FDC somata.

Membrane-stained macrophages with *CD68* were segmented in a similar manner to FDCs, except for object separation (macrophages are basically the largest bystander cells and are spread out), morphological closing was used to improve the completeness of the macrophage segments. Figures 1 and 2 show an example of the segmentation of macrophages.

For all segmented cell types, for each segmented cell, a coordinate pair was computed as the medoid of all pixel coordinates contributing to the respective segment. This means, for all lymph follicles, either from physiological or from lymphoma cases, we obtained a set of corresponding point patterns. Parts of the spatial analysis were made using *Mathematica* (version 11.0.1.0, while the main part was done using *R* (version 3.2.2, The R Foundation for Statistical Computing, Vienna, Austria), in particular using the *R*-package *spatstat* (version 1.42.-2). Simply put, the aim of these analyses was to determine the nature of all point patterns (clustered, random or regular) and to calculate the degree of clustering.

*Functional statistics*

Second order spatial analysis includes functions that are derived from point patterns and respective results can then be related to theoretical distributions (reference null models). A common and simple reference null model is the homogeneous Poisson process (Illian 2008), (Cressie 1993), which generates point patterns of random spatial distribution, also referred to as complete spatial randomness (CSR).

A conventional function is Ripley's $K$-function (B. D. Ripley 1976), which provides information about the spatial dependency of all points over different scales of the pattern. It is defined as

| $K\left( r \right)=\frac{1}{\lambda}E[number of extra points within the distances r of a randomly chosen point]$ | (1) |
| --- | --- |

with $\lambda$ as the point density (number of points per image area) and $E$ denoting the expectation value. The unbiased estimator of the $K$-function is

| $\hat{K}\left( r \right)=\frac{A}{n^{2}}\sum_{i=1}^{n} \sum_{j\neq i}^{n} \frac{I_{r}(u_{ij})}{w_{ij}}$ | (2) |
| --- | --- |

where $n$ is the number of points in the image area $A$, $u_{ij}$ represents the distance between the $i$-th and the $j$-th point, $I_{r}(u_{ij})$ is an indicator function which equals $1$ when$u_{ij}\leq r$ and $0$ otherwise, and $w_{ij}$ is the edge correction factor (Szwagrzyk 1992), (Haase 1995). In principle, a circle of radius $r$ is constructed around each point $i$, the number of other points $j$ within this circle is counted (suppl. Fig. 3; left) and the proportion of the circle that lies within the image area is calculated. These three steps are repeated for all points $i$ and the result is accumulated. Then, $r$ is being incremented by a small amount and the procedure is repeated.

Under the assumption of CSR the expected number of points within the distance $r$ of a point is

| $K_{pois}\left( r \right)=\pi r^{2}$  $K\left( r \right)>\pi r^{2} \overset{\Rightarrow}{} clustering at distance r$  $K\left( r \right)<\pi r^{2} \overset{\Rightarrow}{}dispersion at distance r$ | (3) |
| --- | --- |

$K(r)$ is usually plotted against *r* and deviations from CSR can be observed when $K(r)<\pi r$^2^ (indicating a regular pattern) or $K(r)>\pi r$^2^ (indicating a clustered pattern).

Interpretation of the $K$- function is simplified when it is transformed into its standardized, variance-stabilized version $L(r)$ (Besag 1977):

| $\hat{L}\left( r \right)=\sqrt{\frac{\hat{K}(r)}{\pi}}-r$ | (4) |
| --- | --- |

Under the assumption of CSR the expected number of points within the distance $r$ of a point is

| $L_{pois}\left( r \right)=0$  $L\left( r \right)>0 \overset{\Rightarrow}{} clustering at distance r$  $L\left( r \right)<0 \overset{\Rightarrow}{}dispersion at distance r$ | (5) |
| --- | --- |

$L(r)$ can be plotted against $r$ and deviations from CSR can be observed when $L(r)<0$ (indicating a regular pattern) or $L(r)>0$ (indicating a clustered pattern) (suppl. Fig. 1, lower left).

While $K$- function and $L$- function are of cumulative character and measure the number of points up to a defined distance, the $g(r)$ function (Stoyan 1994):

| $g\left( r \right)=\frac{1}{2\pi r}\frac{dK(r)}{dr}$  $g\left( r \right)>1 \overset{\Rightarrow}{} clustering at interpoint distance of r$  $g\left( r \right)<1 \overset{\Rightarrow}{}dispersion at interpoint distance of r$ | (6) |
| --- | --- |

$g(r)$ is noncumulative and provides information about regularity or clustering at a particular distance. The procedure resembles the calculation of the $K$-function, with the difference being that only those points are counted that are located within a narrow annulus of diameter $d$ at a circle of radius $r$ around each point (suppl. Fig. 3; right). CSR is strictly indicated when $g(r)=1$ holds true, while regularity is suggested if $g(r)<1$, and clustering if $g(r)>1$ (Fig. 7). Sometimes $g(r)$ is also referred to as *pair-correlation* or PC function.

Test of complete spatial randomness

Point patterns can be tested for departure from CSR, whereby CSR acts as a “dividing line“ between regular and clustered patterns (Diggle 2003). Such tests can be categorized into envelope and deviation tests (Szmyt 2014).

Envelope tests (B. D. Ripley 1976) compare the observed summary characteristic of a function (e.g. the *g* function) of a given point pattern to estimates obtained from a simulation of the null model, which uses the estimated parameters in the same image area. Generally, the null model is simulated a number of times, and the extreme values from all simulations $g_{min}(r)$ and $g_{max}(r)$ of estimator $ĝ(r)$ are determined. Extreme values can be plotted together with the estimator $ĝ(r)$ and form the envelopes of $ĝ(r)$. If $ĝ(r)$ is located outside the envelope at a certain distance $r$, the null hypothesis of CSR is rejected for that distance $r$. The "direction" of significant departure from CSR is indicated by the position of the function value relative to the envelope: below the envelope hints at regularity while above the envelope suggests clustering. For each $r$, and in conjunction with the respective point pattern, it could then be discussed why the associated function value is located outside the calculated envelope.

Deviation tests calculate the deviation between the observed function and the expectation under the null hypothesis. For this, the information of the functional summary statistic is transformed into a scalar test statistic. After calculation of the deviation measure the p-value of the deviation test can be estimated. The maximum absolute deviation (MAD) test (B. D. Ripley 1976), (B. Ripley 1977) was used to test point patterns for CSR. The resulting p-values were used to prove or disprove CSR for the individual point patterns and the test statistic was used as a parameter for the degree of deviation from CSR. Test statistics were calculated for $L(r)$ (MADL) and $g(r)$ (MADPC) functions. Another test based on the Monte Carlo simulation is the Diggle-Cressie-Loosmore-Ford (Diggle 2003) test (DCLF test), whose results are similar to the MAD test.

$L(r)$ and $g(r)$ functions from FL and GC were pooled and tested for group differences using the studentized permutation test (Hahn 2012). This test allows the direct comparison of point pattern groups on the basis of their functional summary characteristics without the need for extracting scalar characteristics from the functions and thereby omitting valuable spatial information.

**Validation cohort**The validation cohort consists of ten follicular lymphoma (FL) FFPE samples selected from the files of the Department of Pathology, University Hospital Schleswig-Holstein, Campus Kiel which were stained using the same antibody for PD1 as for fluorescence staining but DAB visualization instead. Bright field images of three neoplastic follicles from each case were captured using an Olympus BX46 and an UC90 camera (Olympus, Hamburg, Germany) and saved in the TIFF format (total of 27 photomicrograph). Digital image analysis was then performed using Tissue Studio Sexta (Definiens AG, Munich, Germany). The TIFF images were uploaded to the program, and the region of interest (ROI) was manually selected to include the neoplastic follicles in each slide. Fragments of brown staining of less than 20 µm2 in area were excluded from analysis. The software outputs data tables that contained the number of positively stained and negatively stained cells in the ROI of each image, as well as the X-Y coordinates of the positively stained cells in terms of pixels in the TIFF image. For each image, the program also saved the original staining and an overlay with coloured and counted nuclei. The X-Y coordinates generated from the above image analysis were used in Ripley’s K-function to generate functional statistics on the distribution of the PD1-stained cells, as described in the previous section.

**Supplementary table 1:** Antibodies used for multi-staining. Multiple clones per target were necessary to be able to built multi-staining panels for each target in combination with Ki67 and Pax5.

| **antibody** | **reactivity** | **company** | **clone/catalogue-no.** | **host** | **dilution** | **staining panel** |
| --- | --- | --- | --- | --- | --- | --- |
| primary | Pax5 | Santa Cruz | sc-1974 | goat | 1:100 | 1, 2, 3, 4 |
| primary | Ki67 | selfgenerated | not applicable | mouse | 1:5, 1:10 | 1 |
| primary | Ki67 | Epitomics | Cat4203-1 | rabbit | 1:100 | 2,3,4 |
| primary | CD3 | NeoMarkers | RM-9107-S | rabbit | 1:100 | 1 |
| primary | PD1 | Abcam | NAT | mouse | 1:50 | 2 |
| primary | CD21 | NovoCastra | 2G9 | mouse | 1:10 | 3 |
| primary | CD68 | Cell Signaling | D4B9C | rabbit | 1:200 | 4 |

**Supplementary figures**


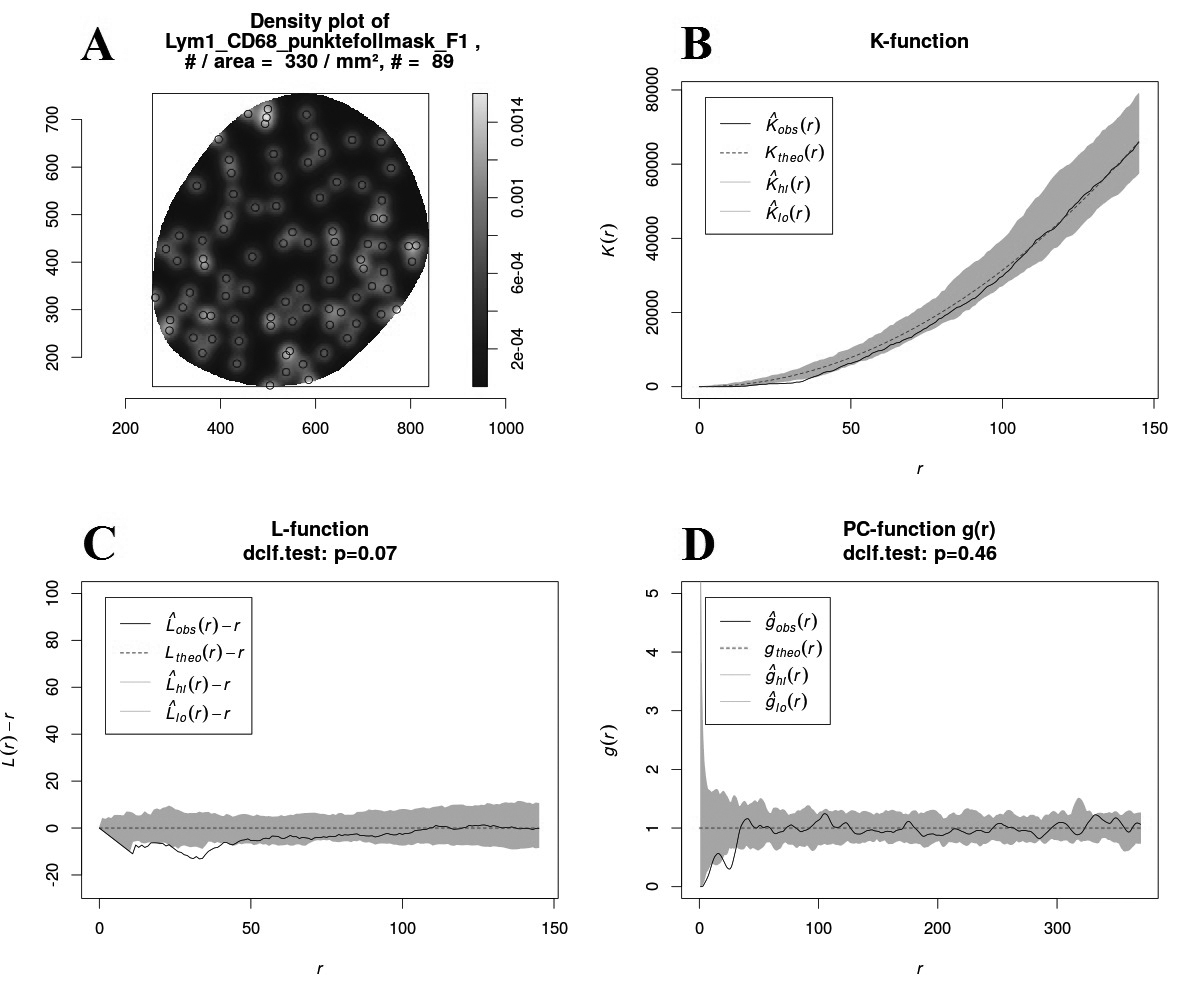


**Supplementary figure 1:** Density plot (A), $K$- function (B), $L$- function (C) and $PC$- function (D). In this figure simulation-based computed envelopes for the radial distribution functions ($K$-, $L$-, and $PC$- function) are depicted. For $L$- and $PC$- functions respective test statistics were computed (DCLF test). The null hypothesis is CSR. Assuming a significance level of 0.05, both parts C and D indicate that the null hypothesis cannot be rejected.

**
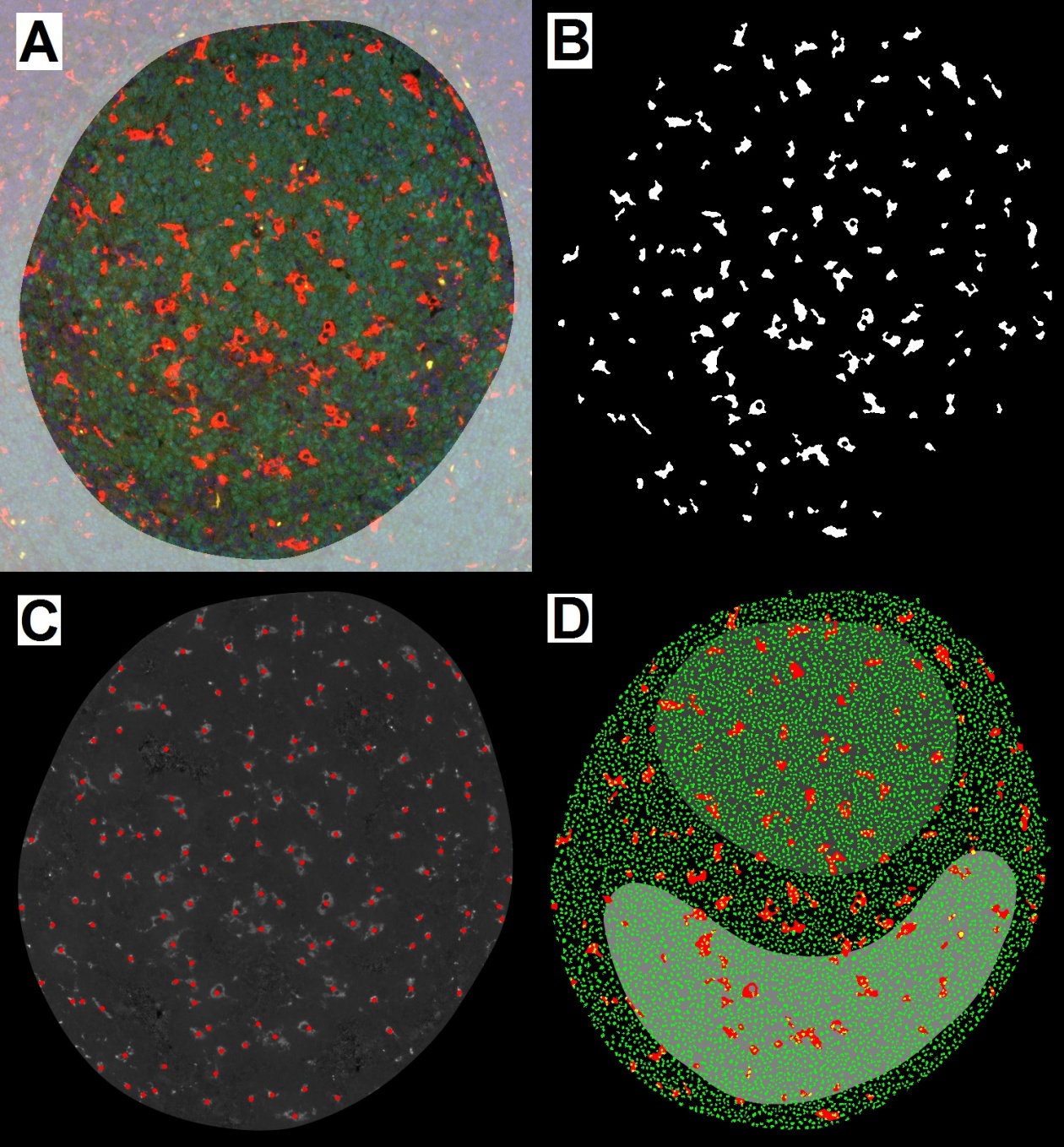
**

**Supplementary Figure 2:** Segmentation process for *CD68* in a physiological germinal center (GC). **A**: Original staining, GC high labeled (CD68 red, Pax5 green, DAPI blue). **B**: Bernsen filter and the binarization. **C**: Macrophages identified by *CD68* (red) indicated by a red dot for each cell. **D**: Macrophages (red) B cells (green) and the division into a light and a dark zone by *Ki67* staining (not shown).

**
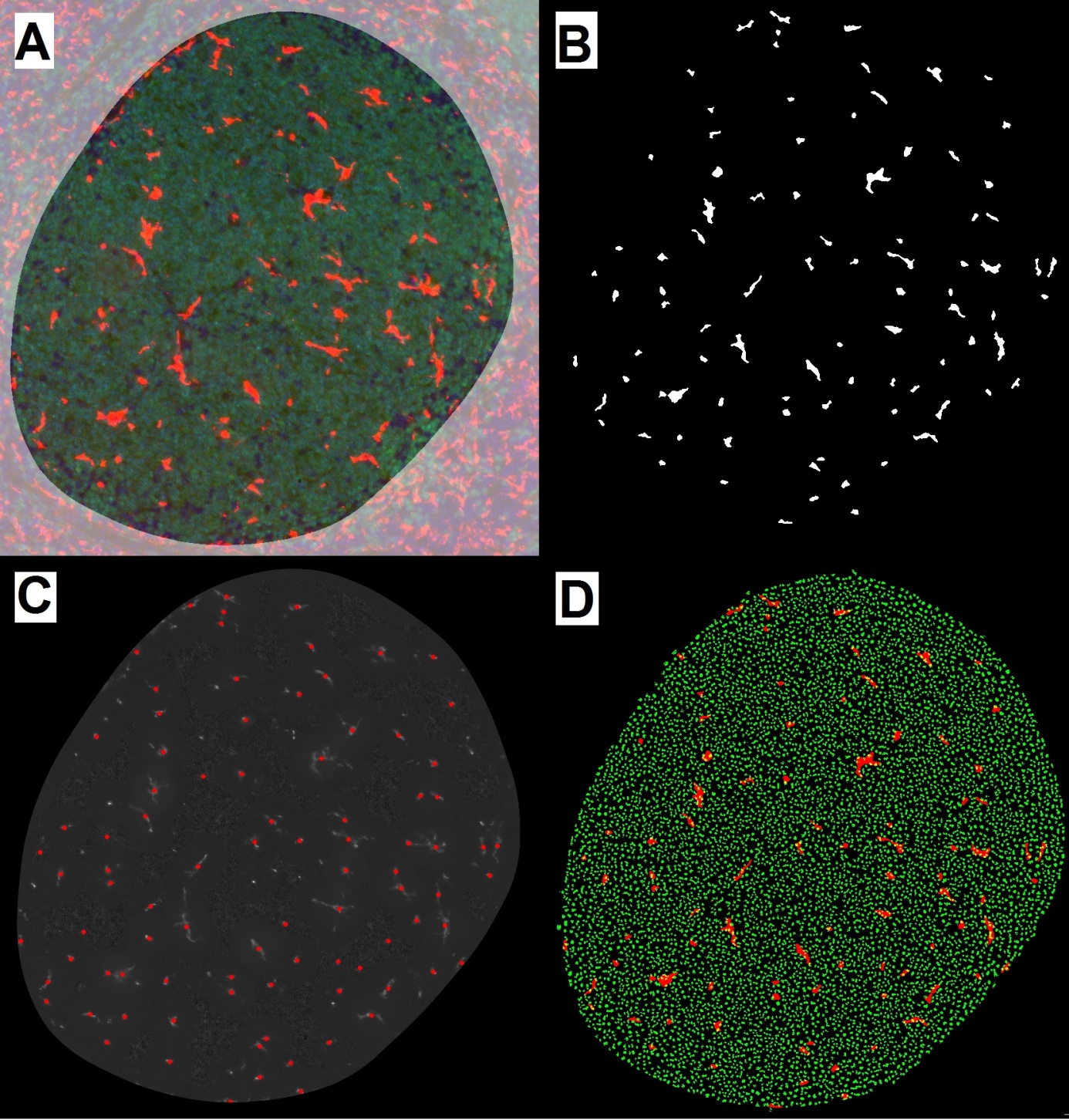
**

**Supplementary Figure 3:** Segmentation process for *CD68* in a neoplastic follicle of a follicular lymphoma. **A**: Original staining, malignant germinal center labeled (CD68 red, Pax5 green, DAPI blue). **B**: Bernsen filter and the binarization. **C**: Macrophages identified by *CD68* (red) indicated by a red dot for each cell. **D**: Macrophages (red) B cells (green).

**
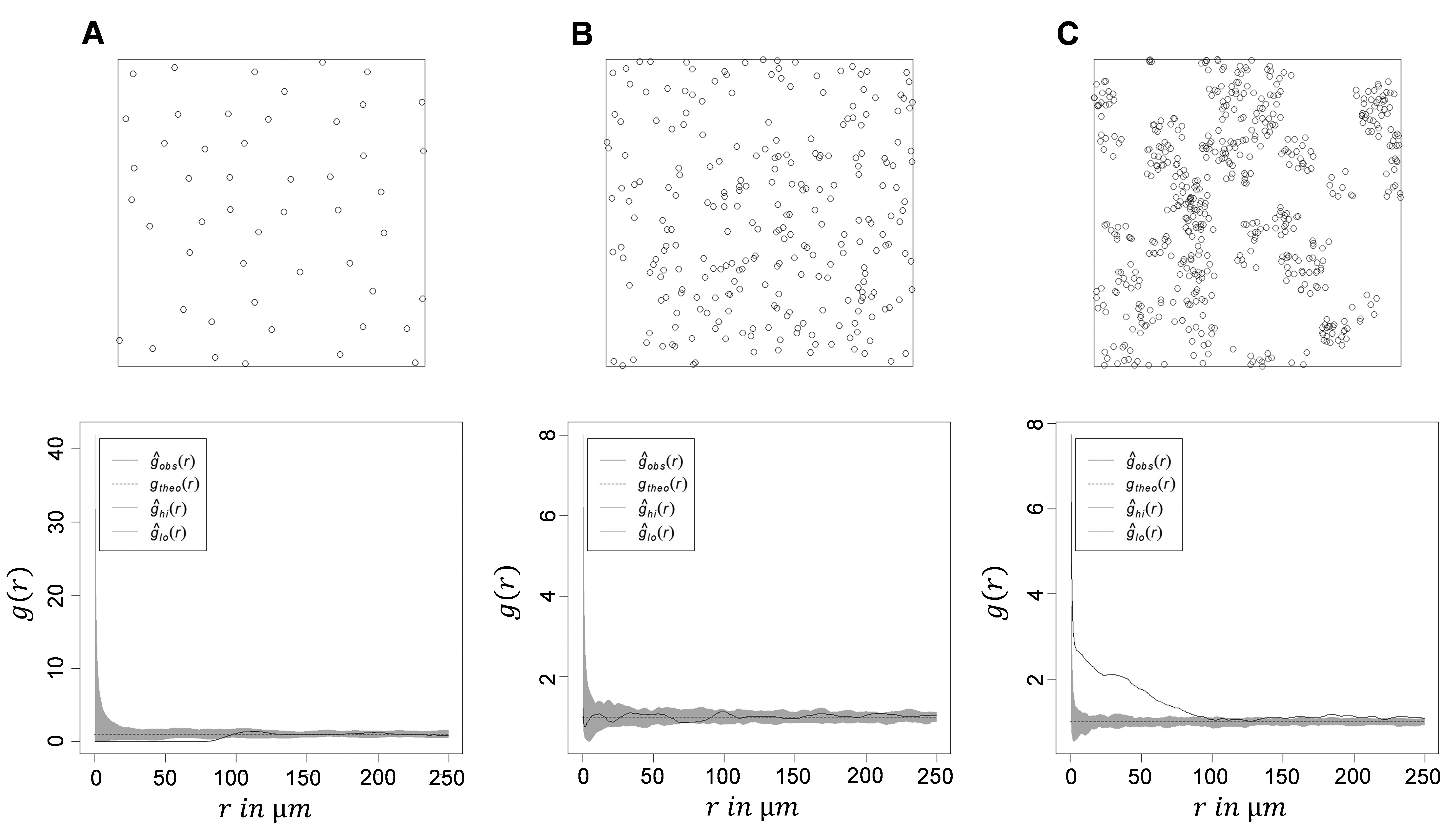
**

**Supplementary Figure 4**: Virtual examples of the distribution pattern of cells (upper panel) and a point-pattern analysis employing Ripley's function (lower panel) for regular pattern (A), complete spatial randomness (CSR, B) and clustering (C). On the x-axis r indicates the radius around any given cell of a certain type, such as macrophages, follicular dendritic cells, T cells of T_FH_ type. On the y-axis g(r) indicates the pair-correlation function applied to all cells of that type. The plot show functions (black lines), computed envelopes (light grey areas) and assumption of CSR (dashed lines) for the respective functions. Functions leaving the envelope indicate significant departure from CSR at the respective distance r. Regularity is suggested if g(r)<1 (A). If g(r)=1, CSR can be assumed (B). g(r)>1 indicates clustering. The example in C indicates clustering of cells in a radius of 1-75 µm.





**Supplementary Figure 5:** Validation cohort staining for PD1 (n=27 images from n=9 follicular lymphomas). The g(r) function for PD1 cell distribution in follicular lymphoma shows clustering in a radius of 5-10 µm (dark blue line, range indicated by light blue lines) which is absent in whole follicles of tonsils (dark red line, range indicated by pink lines).

# Literature

Bernsen, J. "Dynamic thresholding of gray level images." *Proceedings of the International Conference on Pattern Recognition (ICPR '86)*, 1986.

Besag, J. "Discussion of Dr Ripley's paper." *Journal of the Royal Statistical Society*, 1977: 193–195.

Cressie, N.A.C. *Statistics for spatial data.* J. Wiley, 1993.

Diggle, PJ. *Statistical Analysis of Spatial and Spatio-Temporal Point Patterns.* Chapman and Hall/CRC, 2003.

Haase, P. "Spatial pattern analysis in ecology based on Ripley’s K-function: introduction." *Journal of Vegetation Science*, 1995: 575–582.

Hahn, U. "A studentized permutation test for the comparison of spatial point patterns." *Journal of the American Statistical Association*, 2012: 754–764.

Illian, J. and Penttinen, P.A. and Stoyan, H. and Stoyan, D. *Patterns, Statistical Analysis and Modelling of Spatial Point.* Wiley, 2008.

Marr, D. and Hildreth, E. "Theory of Edge Detection." *Proceedings of the Royal Society of London B: Biological Sciences*, 1980.

Meyer, F. "Contrast features extraction." *Jean-Louis Chermant (ed.): Quantitative Analysis of Microstructures in Materials Science, Biology and Medicine*, 1996: 374-380.

Ohser, J. "On estimators for the reduced second moment measure of point processes." *Mathematische Operationsforschung und Statistik, series Statistics*, 1983: 14, 63 - 71.

Otsu, Nobuyuki. "{A} {T}hreshold {S}election {M}ethod from {G}ray-Level {H}istograms." *IEEE Transactions on Systems, Man and Cybernetics*, 1979: 62--66.

Ripley, B. D. "The second-order analysis of stationary point processes." *Journal of Applied Probability*, 1976: 255--266.

Ripley, B.D. "Modelling spatial patterns (with discussion)." *Journal of the Royal Statistical Society*, 1977: 172 – 212.

Stoyan, D. and Stoyan, H. *Fractals, random shapes, and point fields: methods of geometrical statistics.* Wiley, 1994.

Szmyt, J. "Spatial statistics in ecological analysis: From indices to functions." *Silva Fennica*, 2014.

Szwagrzyk, J. "Small scale spatial patterns of trees in mixed pine-beech forests." *Forest*, 1992: 301–315.
